# Galacto-Oligosaccharides as an Anti-Infective and Anti-Microbial Agent for Macrolide-Resistant and -Sensitive *Mycoplasma pneumoniae*

**DOI:** 10.3390/pathogens12050659

**Published:** 2023-04-28

**Authors:** Hongzhen Zhu, Yang Cai, Lisa J. M. Slimmen, Adrianus C. J. M. de Bruijn, Annemarie M. C. van Rossum, Gert Folkerts, Saskia Braber, Wendy W. J. Unger

**Affiliations:** 1 Laboratory of Pediatrics, Department of Pediatrics, Erasmus MC, University Medical Centre Rotterdam, Sophia Children’s Hospital, 3015 GD Rotterdam, The Netherlands; h.zhu@erasmusmc.nl (H.Z.);; 2 Division of Pharmacology, Utrecht Institute for Pharmaceutical Sciences, Faculty of Science, Utrecht University, 3584 CG Utrecht, The Netherlands; 3 Department of Pharmacology, Jiangsu Provincial Key Laboratory of Critical Care Medicine, School of Medicine, Southeast University, Nanjing 210009, China; 4 Department of Pediatrics, Division of Pediatric Infectious Diseases and Immunology, Erasmus MC, University Medical Center Rotterdam, Sophia Children’s Hospital, 3015 GD Rotterdam, The Netherlands

**Keywords:** *Mycoplasma pneumoniae*, antibiotic resistance, galacto-oligosaccharides, bactericidal effects, anti-infective, human epithelial cells

## Abstract

The worldwide increase in the incidence of antibiotic resistance of the atypical bacterium *Mycoplasma pneumoniae* (MP) challenges the treatment of MP infections, especially in children. Therefore, alternative strategies for the treatment of MP infections are warranted. Galacto- and fructo-oligosaccharides (GOS and FOS) are a specific group of complex carbohydrates that were recently shown to possess direct anti-pathogenic properties. In this study, we assessed whether GOS and FOS exert anti-microbial and anti-infective effects against MP and, especially, macrolide-resistant MP (MRMP) in vitro. The MIC values of GOS for MP and MRMP were 4%. In contrast, the MIC values of FOS for both MP and MRMP were 16%. A time-kill kinetic assay showed that FOS possess bacteriostatic properties, while for GOS, a bactericidal effect against MP and MRMP was observed after 24 h at a concentration of 4x MIC. In co-cultures with human alveolar A549 epithelial cells, GOS killed adherent MP and MRMP and also concentration-dependently inhibited their adherence to A549 cells. Further, GOS suppressed (MR)MP-induced IL-6 and IL-8 in A549 cells. None of the aforementioned parameters were affected when FOS were added to these co-cultures. In conclusion, the anti-infective and anti-microbial properties of GOS could provide an alternative treatment against MRMP and MP infections.

## 1. Introduction

*Mycoplasma pneumoniae* (MP) is one of the most prevalent bacterial species causing community-acquired pneumonia (CAP) in children and adults [1,2]. Despite the fact that most MP-induced CAPs present as mild disease, severe cases can also occur and require hospitalization and antibiotic treatment [3,4,5]. Since MP lacks a cell wall, MP infections are treated with macrolides, tetracyclines and fluoroquinolones, which target protein synthesis and DNA replication [6]. For young children, macrolides are the first choice of therapy due to concerns regarding the toxicity of tetracyclines and fluoroquinolones [7,8]. However, the accurate diagnosis of MP infections is difficult, which may lead to macrolide mis- and/or overuse in pediatric patients. Additionally, this causes the emergence of macrolide resistance in MP [9,10,11].

The percentage of macrolide-resistant MP (MRMP) has increased rapidly among pediatric patients due to the extensive use of erythromycin (ERY), especially in Asia, where resistance rates are as high as 90% [11,12,13,14]. MRMP is a relevant clinical problem, as patients with MRMP infections have more serious symptoms and a higher chance of extrapulmonary complications and require longer hospitalization than those with macrolide-sensitive MP infections [7]. Furthermore, as asymptomatic carriage can result in infection in the same host, the carriage of MRMP can be particularly dangerous for immunocompromised individuals [15]. Together, it is evident that alternative anti-microbial therapies for the treatment of MRMP and MP infections are needed to keep treatment sustainable [13,16,17,18].

Non-digestible oligosaccharides (NDOs), including human milk oligosaccharides (HMOs) and galacto- and fructo-oligosaccharides (GOS and FOS), are a group of complex carbohydrates with potential direct and indirect anti-pathogenic functions [19]. When administered orally, these NDOs can strengthen gut health through positive effects on the gut microbiome and the stimulation of immune homeostasis [20,21,22,23,24,25]. Recently, the direct anti-pathogenic effects of GOS and FOS have gained interest. In vitro studies showed that GOS prevented the adhesion of *Escherichia coli* and *Mannheimia haemolytica* to the intestinal and respiratory epithelium, respectively [26,27]. It has been speculated that GOS act as soluble decoy receptors that bind either to structures on the pathogen or to the target receptors on the host epithelial cell surface. Additionally, both GOS and FOS inhibit the growth and biofilm formation of bacteria such as *Pseudomonas aeruginosa* strain PAO1 [28]. Based on these studies, it is clear that GOS and FOS possess anti-microbial capacity on different bacteria; however, whether they also act on atypical bacteria such as MP and whether they are effective in controlling MRMP in particular is unknown.

In this study, we therefore investigated whether GOS and FOS affect the growth and viability of MP and, especially, MRMP in vitro, as well as their adherence to and inflammatory response induced in alveolar A549 epithelial cells. 

## 2. Materials and Methods

### 2.1. Bacterial Strains

Both macrolide-sensitive and macrolide-resistant *M. pneumoniae* strains were used in this study. The macrolide-sensitive strains include the reference strains *M. pneumoniae* M129 (subtype 1; ATCC 29342) and FH (subtype 2, ATCC 15531) as well as the clinical isolates A103 and H010 (derived from symptomatic CAP patients) [29]. The macrolide-resistant strains *M. pneumoniae* A58, M688/98, T79 and P05/132 were clinical isolates derived from symptomatic CAP patients [29,30]. *M. pneumoniae* strains were cultured in SP4 medium (1.4% Difco PPLO broth supplemented with 0.15% Difco TC Yeastolate, UF, 1.4% glucose, 20% horse serum, 0.02 mg/mL phenol red, 1000 U/mL penicillin, 500 U/mL polymyxin B, 3 μg/mL amphotericin B and 5 μg/mL voriconazole; pH 7.8–8.0) at 37 °C and 5% CO_2_ until a color change of the medium. Then, bacteria were harvested, washed and concentrated in fresh SP4 medium to 1 × 10^9–10^ CFU/mL. Aliquots were stored at −80 °C until use.

### 2.2. NDOs and Antibiotics

The NDOs, GOS (Vivinal GOS syrup, Friesland Campina Ingredients, Amersfoort, The Netherland) and FOS (Frutalose OFP, Sensus, The Netherlands) were used. GOS and FOS were dissolved in fresh SP4 medium or RPMI 1640 culture medium to reach final concentrations ranging from 2% to 16%. ERY (Sigma-Aldrich, St. Louis, MO, USA) was dissolved in fresh SP4 culture medium or RPMI 1640 culture medium to reach final concentrations ranging from 0.001 to 1.024 μg/mL.

### 2.3. Determination of Minimum Inhibitory Concentration (MIC) and Minimum Bactericidal Concentration (MBC)

The MIC values of GOS and FOS were determined by the color change of the medium, as described previously, with slight modifications [31]. In brief, 10^4^ CFU MP or MRMP were cultured in 250 µL SP4 medium at 37 °C and 5% CO_2_ in 96-well flat-bottom sterile polystyrene plates (Greiner Bio-one) in the presence of increasing concentrations of GOS or FOS, using ERY as the control. After incubation for MIC determination, 50 µL of the culture medium was transferred from the MIC plate to fresh SP4 medium. Subsequently, 100 µL of diluted supernatants was inoculated onto PPLO agar plates and cultured for 2 weeks at 37 °C, and the MBC values of GOS and FOS were determined by CFU counting. In addition, the additive effects of GOS and ERY were tested as described above. The kinetics of the bactericidal effect of GOS were tested after culturing for 6 h and 24 h.

### 2.4. Human Respiratory Epithelial Cell Culture 

The human respiratory epithelial cell line A549 (ATCC, CCL-185) was cultured at 37 °C/5% CO_2_ in RPMI-1640 medium supplemented with 10% heat-inactivated fetal bovine serum and without antibiotics.

### 2.5. Lactate Dehydrogenase (LDH) Assay

To measure the potential toxic effects of NDOs on alveolar A549 cells, the cells were seeded at a density of 5 × 10^5^ cells/mL in 24-well plates and cultured overnight to reach 80% confluence. The next day, the medium was replaced by fresh culture medium containing different concentrations of NDOs (2-16%). After 24 h of incubation, the supernatants were harvested to evaluate LDH leakage. LDH was measured using the CyQUANT LDH Cytotoxicity Assay kit (Invitrogen), according to the manufacturer’s instructions.

### 2.6. MP and MRMP Adhesion to A549 Cells

Confluent monolayers of A549 cells were co-cultured for 4 h or 24 h with 10^8^ CFU MP (M129) or MRMP (A58) in the presence of different concentrations of GOS or FOS. Subsequently, the supernatants were harvested, and A549 cells were lysed using milliQ. Both the lysed cells (revealing the number of adherent bacteria) and the culture supernatants (revealing the number of viable, non-adherent bacteria) were diluted in SP4 medium and inoculated onto PPLO agar plates to evaluate the antibacterial capacity of GOS. To investigate the therapeutic effect of NDOs, confluent monolayers of alveolar A549 cells were incubated for 4 h with 10^8^ CFU MP; after removing the non-adherent MP by washing, different concentrations of GOS or FOS were added, and the cells were cultured further. After 20 h, the A549 cells were lysed, diluted in SP4 medium and inoculated onto PPLO agar plates to evaluate the microbicidal capacity of NDOs for adherent (MR)MP. As controls, the medium or ERY were added to the cultures. The anti-adhesion and bactericidal effects were determined by CFU counting.

### 2.7. ELISA

Interleukin (IL)-6 and IL-8 concentrations in the supernatants were measured by ELISA using specific antibody pairs (Thermo Fisher Scientific, Waltham, MA, USA), according to the manufacturer’s instructions. The absorbance was measured at OD450 nm using a microplate reader (SpectraMax iD3, Molecular Devices, San José, CA, USA).

### 2.8. Statistical Analysis

Data were reported as the mean values ± SEM of at least three independent experiments (*n* = 3) routinely performed in triplicate. Statistical analysis was performed using GraphPad Prism 9.0 (GraphPad Software Inc., San Diego, CA, USA). Statistically significant differences between groups were determined by one-way ANOVA with post hoc Dunnett’s multiple comparisons test. The results were considered statistically significant when *p* < 0.05.

## 3. Results

### 3.1. Inhibition of MP and MRMP Growth by GOS and FOS

To evaluate whether GOS and FOS can inhibit the growth of MP and MRMP, we determined the MIC values based on the color change of the SP4 medium [31]. As shown in Table 1, the MIC values of GOS for all MP and MRMP strains were 4%. In contrast, FOS only effectively inhibited the growth of MP and MRMP strains at 16%. The MIC values of ERY for MP and MRMP were 0.032 μg/mL and 256 μg/mL, respectively. 

### 3.2. Bactericidal Effect of GOS and FOS on MP and MRMP

**Figure 1:**
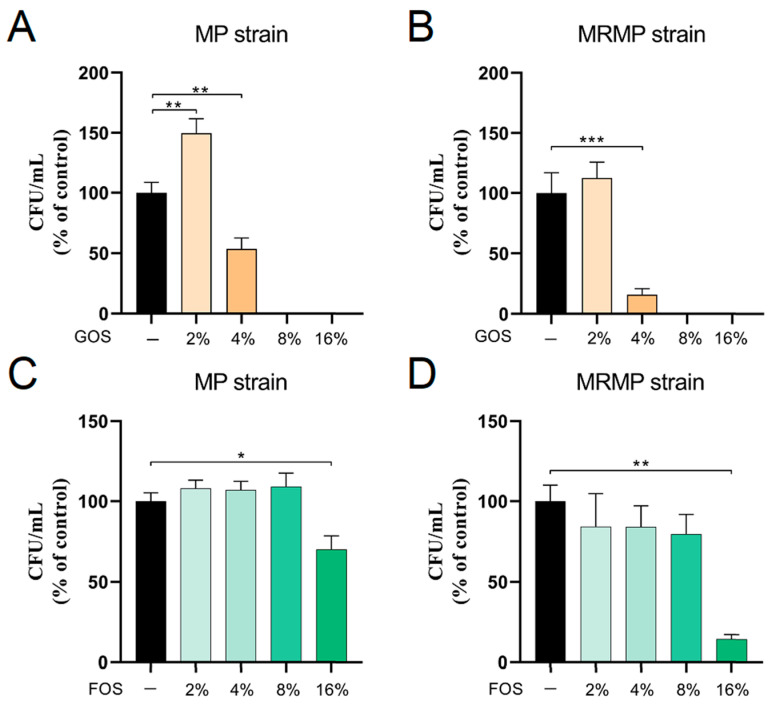
The bactericidal effect of GOS against MP and MRMP strains. (**A**,**C**) MP and (**B**,**D**) macrolide-resistant MP (MRMP) strains were co-cultured with indicated concentrations of GOS or FOS in triplicate in 96-well plates. After 5 days, culture supernatants were re-suspended and inoculated onto PPLO agar plates, and CFUs were counted. The mean CFU/mL ± SEM are shown as the % of control. Data of at least three independent experiments. All measurements consist of three technical replicates. * *p* < 0.05, ** *p* < 0.01 and *** *p* < 0.001 (One-way ANOVA followed by Dunnett’s multiple comparison test).

To investigate whether GOS and FOS also show bactericidal effects against MP and MRMP (A58 strain), the survival of MP and MRMP was determined by CFU plating after MIC assays. As shown in Figure 1, compared to the growth control, the survival of MP and MRMP at 4% GOS was reduced by, respectively, 50% and 80% (Figure 1A,B). No MP and MRMP survived at 8% and 16% GOS, whereas the amounts of MP and MRMP were slightly increased at 2% GOS. In contrast, FOS did not show a bactericidal effect on the MP and MRMP; however, the viability of MP and MRMP at 16% FOS was decreased (Figure 1C,D).

**Figure 2:**
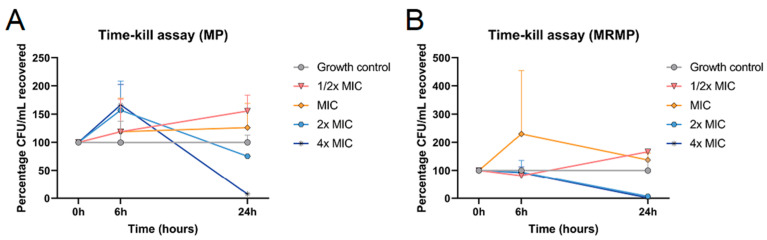
Bactericidal activity of GOS against MP and MRMP strains within 24 h. Bactericidal activity of GOS against MP and MRMP strains within 24 h. (**A**) MP and (**B**) MRMP strains were cultured in the presence of indicated concentrations of GOS in triplicate in 96-well plates. After culture for 6 h and 24 h, cells were re-suspended and inoculated onto PPLO agar plates, and the CFUs were determined. All CFU counts are normalized against the medium growth control. MIC is 4% GOS. The mean CFU/mL ± SEM are shown as the percentage CFU/mL recovered. Representative results from three independent experiments.

Since MP grow quite slowly, these initial MBC assays were started on day 5 of the culture. However, in time-kill kinetics experiments, we assessed that GOS affected the viability of MP and MRMP at 24 h. No bactericidal effects were observed within 6 h; in fact, MP and MRMP were growing. As shown in Figure 2A, the survival of MP was decreased by 24% and 92% after 24 h of culturing with 8% and 16% GOS, respectively, and the survival of MRMP was already decreased by 93% and 98%, respectively, when compared to the growth control cultures (Figure 2B). 

### 3.3. GOS Act Additive with ERY on the Viability of MP but Not MRMP

**Figure 3:**
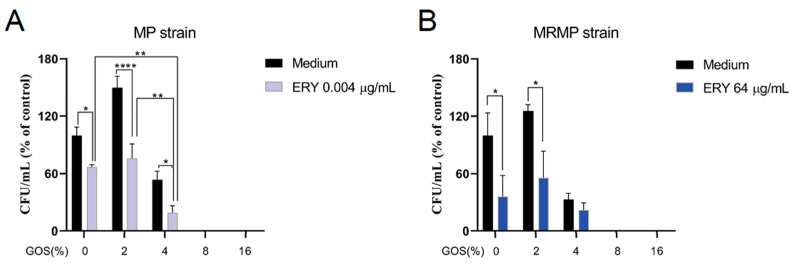
Additive effects of GOS and ERY on the viability of MP but not MRMP. Additive effects of GOS and ERY on the viability of MP but not MRMP. (**A**) MP and (**B**) MRMP strains were cultured in the presence of GOS combined with ERY and compared to wells containing ERY only. Growth controls contained medium only. When a color change of the medium was noted, bacterial cells were re-suspended and inoculated onto PPLO agar plates, and CFUs were determined. The mean CFU/mL ± SEM are shown as % of medium control. * *p* < 0.05, ** *p* < 0.01 and **** *p* < 0.0001 (One-way ANOVA followed by Dunnett’s multiple comparison test).

The finding that GOS decreases the viability of the slow-growing MP within 24 h and also acts on MRMP at the same concentration as on MP strongly suggests that GOS act via a different mechanism than macrolides and that these compounds could potentially be combined for treatment [32]. We therefore combined different GOS concentrations with ERY. These assays revealed additive effects of 4% GOS when combined with a suboptimal concentration of ERY (i.e., 0.004 µg/mL for MP), as the survival of MP in these cultures was decreased by 80% and 34% compared to the growth control- and ERY-treated cultures, respectively (Figure 3A). In contrast, additive effects of GOS combined with 64 µg/mL ERY were not found on MRMP (Figure 3B).

### 3.4. GOS Kills MP and MRMP That are Adherent to Respiratory Epithelial Cells

Next, we assessed whether the bactericidal effects of GOS on MP and MRMP observed in planktonic cultures also occur when these bacteria are present on respiratory epithelial cells, the first host cells that are encountered during an infection. We used confluent A549 epithelial cultures. Testing the direct effects of NDOs on A549 cells revealed that GOS, at concentrations ≥ 16%, affected the A549 viability, as a lower survival rate after 24 h of incubation compared to untreated cultures was observed (Figure S1A). Although FOS did not show cytotoxic effects on A549 cells at these concentrations (Figure S1B), in the next experiments, the maximum concentrations of either NDO used was 8%. 

**Figure 4:**
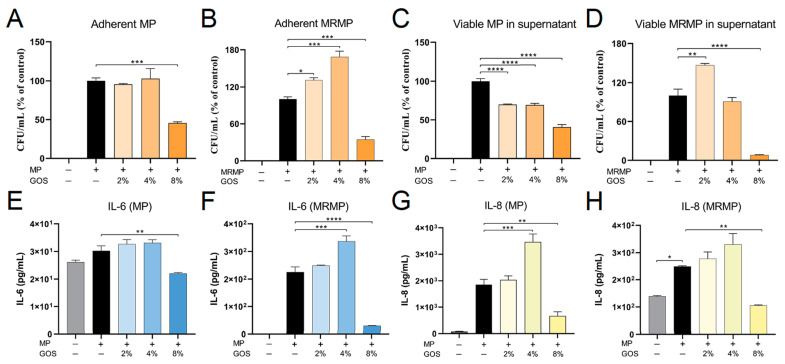
GOS kill MP and MRMP that are adherent to respiratory epithelial cells. MP or MRMR first adhered to respiratory epithelial cells by co-culturing for 4 h. After the removal of non-adherent bacteria, co-cultures were treated with indicated concentrations of GOS. Growth control cultures were treated with ERY or with only the medium. After overnight culture, the supernatants were collected, and epithelial cells were lysed. Both cells (**A**,**B**) and culture supernatants (**C**,**D**) were inoculated onto PPLO agar plates, and CFUs were determined. The mean CFU/mL ± SEM are shown as the % of control. The supernatants were collected, and IL-6 (**E**,**F**) and IL-8 (**G**,**H**) levels in the supernatant were measured by ELISA. Bars represent the mean cytokine concentration ± SEM. * *p* < 0.05, ** *p* < 0.01, *** *p* < 0.001 and **** *p* < 0.0001 (One-way ANOVA followed by Dunnett’s multiple comparison test).

In patients, the MP or MRMP is already adherent to and/or infecting the airway epithelial cells when consulting a clinician because of respiratory tract infection symptoms. To mimic this situation, the bacteria were co-cultured with A549 cells for 4 h, after which the non-adherent bacteria were washed away and GOS was added. The analysis of adhering MP or MRMP 20 h later showed a significant reduction in the number of adhering bacteria at 8% GOS compared to the untreated wells. The amounts of adherent MP and MRMP were decreased by 54% and 65%, respectively (Figure 4A,B). The assessment of the corresponding culture supernatants revealed a significant reduction in viable MP and MRMP. The viability of MP and MRMP was decreased by 59% and 91% compared to the untreated wells (Figure 4C,D). Additionally, we observed that the (MR)MP-induced secretion of IL-6 and IL-8 was suppressed by 8% GOS. Compared to untreated MP and MRMP cultures, IL-6 levels were decreased by 92% and 86%, respectively, in GOS-treated cultures (Figure 4E,F), while IL-8 concentrations at 8% GOS were decreased by 64% and 79%, respectively (Figure 4G,H). At 2% or 4% GOS, the MRMP-induced secretion of IL-6 and IL-8 was increased, reflecting the higher number of MRMP bacteria adherent to the alveolar A549 cells. No effect on adhesion or inflammatory cytokine production was observed in ERY-treated cultures (Figure S2). 

### 3.5. GOS Prevents the Adhesion of MP and MRMP to A549 Cells

**Figure 5:**
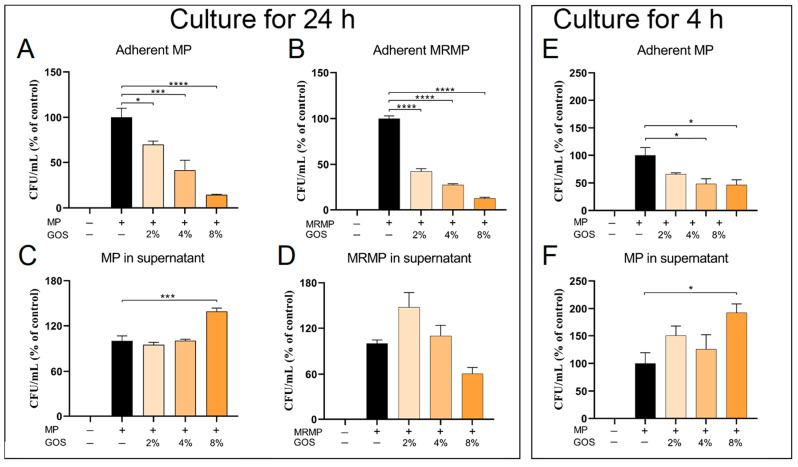
GOS concentration-dependently inhibit MP & MRMP adhesion to epithelial cells and kill MP & MRMP. MP or MRMP and GOS at indicated concentrations were added to epithelial cell monolayers. After 24 h (**A**–**D**) or 4 h (**E**,**F**), supernatants were collected, and epithelial cells were lysed. Both cells (**A**,**B**,**E**) and culture supernatants (**C**,**D**,**F**) were inoculated onto PPLO agar plates, and CFUs were determined. The mean CFU/mL ± SEM are shown as the % of control. * *p* < 0.05, *** *p* < 0.001 and **** *p* < 0.0001 (One-way ANOVA followed by Dunnett’s multiple comparison test was used for statistical analysis).

Interference in the process of the MP carriage will decrease the potential induction of disease, as the carriage can result in infection in the same host or, upon horizontal transfer, in other hosts [30,33]. MP carriage starts with the adhesion of the bacteria to the respiratory epithelium. We therefore evaluated whether GOS and FOS interfered with the epithelial adherence of MP and MRMP by adding bacteria and NDOs simultaneously to epithelial cells. Compared to the untreated cultures, the amounts of MP and MRMP adherent to A549 cells were significantly decreased in cultures treated for 24 h with GOS. The number of adherent MP and MRMP decreased proportionally with increasing concentrations of GOS (Figure 5A,B). However, the assessment of the number of alive MP and MRMP in the corresponding culture supernatants indicated that MRMP but not MP bacteria were killed (Figure 5C,D). To assess whether the observed reduction in adherent bacteria was due to interference with the adhesion process or due to killing of bacteria, we subsequently assessed these parameters after 4 h of incubation, as we previously found that GOS at a concentration of 4% was ineffective in killing MP and MRMP in such a short timeframe (Figure 2). Indeed, after 4 h of culture, the amounts of MP adherent to the A549 cells were significantly decreased in GOS-treated cultures, but the number of alive MP in the culture supernatants showed that the bacteria were not killed within 4 h (Figure 5E,F). In contrast to GOS, the addition of FOS did not block the adhesion of the MP and MRMP bacteria (Figure S3). 

**Figure 6:**
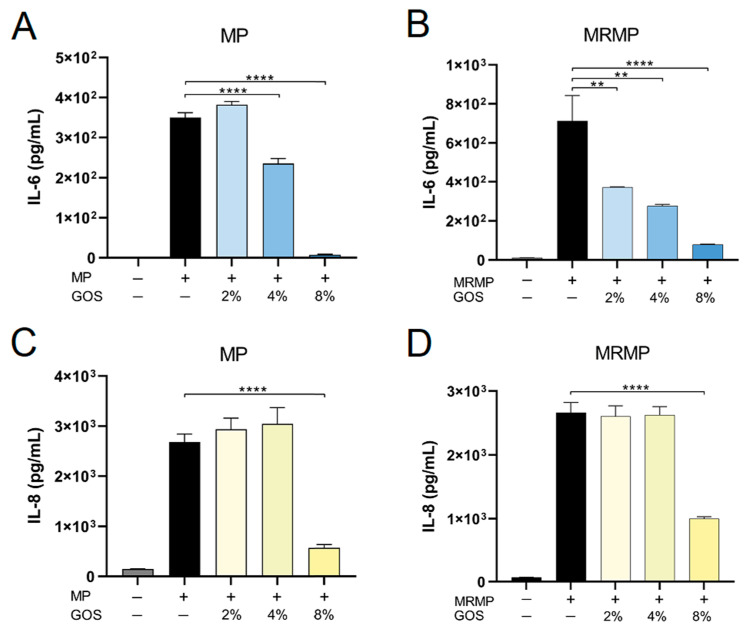
GOS concentration-dependently inhibit IL-6 and IL-8 by MP & MRMP induced on epithelial cells. MP or MRMP and GOS at indicated concentrations were added to epithelial cell monolayers. After 24 h, supernatants were collected, and IL-6 (**A**,**B**) and IL-8 (**C**,**D**) levels were measured by ELISA. Bars show the mean cytokine concentration ± SEM. ** *p* < 0.01, and **** *p* < 0.0001 (One-way ANOVA followed by Dunnett’s multiple comparison test was used for statistical analysis).

In alignment with the prevented adhesion, IL-6 levels in MP and MRMP cultures containing 4% and 8% GOS were lower than those in untreated cultures (Figure 6A,B). The MP- and MRMP-induced secretion of IL-8 was only prevented by GOS at a concentration of 8% (Figure 6C,D). 

## 4. Discussion

In the present study, we demonstrated that GOS have bactericidal effects against the atypical respiratory pathogen MP and, specifically, against MRMP in vitro. These effects were also observed when MP bacteria were adherent on respiratory epithelial cells in vitro. Importantly, we have demonstrated that GOS could also prevent the adhesion of MP and MRMP onto epithelial cells, thereby suppressing MP- and MRMP-induced epithelial inflammation.

GOS showed both bacteriostatic and bactericidal effects on MRMP and MP strains in planktonic cultures. The bacteriostatic effects of GOS may be exerted by interfering with the MP P1 and P30 proteins. Both P1 and P30 are located in the terminal organelle and are involved in MP cell division and motility [34,35,36]. A previous study on alternative treatments for MP showed that the Platycodon grandiflorus-derived compound Platycodon D significantly inhibited MP growth by decreasing the expression of its proteins P1 and P30 [37]. In our GOS-treated cultures, we did not observe effects on *P1* levels, which suggests that GOS act via a different mechanism than Platycodon D to inhibit MP growth. Similar to GOS, FOS also inhibited MP and MRMP growth, although very high concentrations were required to obtain that effect. The need for such high concentrations of FOS may be dependent on the fact that, here, FOS were tested in an in vitro model that does not include other cells that could metabolize FOS. Indeed, the control of *E. coli* in the intestines of pigs by FOS appears to be via its hydrolysis into metabolites such as short-chain fatty acids and acetate [38]. These metabolites, and acetate in particular, caused a lowering of intestinal pH, which is detrimental to the growth of pathogenic bacteria such as *E. coli* [39,40,41].

Besides bacteriostatic effects, GOS also clearly possess bactericidal properties. The viability of MP and MRMP in planktonic cultures was reduced by 90% upon incubation with GOS. Moreover, the bactericidal effects of GOS were even more prominent against MRMP and MP that adhered onto alveolar epithelial monolayers in vitro. The strong reduction in the numbers of adherent MP and MRMP together with the observation that the viability of MP and MRMP in the supernatants of these cultures was reduced by 50% indicates that GOS directly affects the viability of (MR)MP. A recent study showed that GOS killed the bovine pathogen *M. haemolytica* via the destruction of its membranes [27]. 

The observation that GOS also act on MRMP at the same concentration as on macrolide-sensitive MP suggests that GOS and macrolides act via different mechanisms [42,43,44]. This makes it ideal to combine these two compounds for the treatment of both MP and MRMP infections, as it would allow for the use of lower concentrations of the macrolide. We observed that GOS potentiated the activity of ERY against MP: a suboptimal concentration of ERY was significantly more effective in killing MP when combined with 4% GOS, indicating an additive effect. GOS thus might provide new opportunities for reducing ERY over-use and thereby reduce the risk of inducing macrolide resistance. Macrolide resistance is a major issue in MP treatment in clinics, as the efficacy of macrolide treatment is only 22.7% for MRMP in comparison to a 91.5% efficacy for macrolide-sensitive MP [7]. While an additive effect of ERY combined with 4% GOS was found for MP, this was not found for MRMP. The underlying events leading to these differences between MP and MRMP in terms of sensitivity for ERY-GOS are the subject of future studies. 

Using a set-up in which GOS and MP/MRMP were simultaneously added to the epithelial barriers, we found that GOS, but not FOS, have an anti-adhesive effect against MP and MRMP. GOS could act as a decoy receptor to MP adhesins or competitively bind to A549 cells to prevent the MP and MRMP adhesion. Such decoy activities of GOS and other NDOs have been shown to inhibit the epithelial adhesion of *E. coli*, *Salmonella typhimurium, M. haemolytica* and *Cronobacter sakazakii * [26,27]. The cytoadherence of MP to the surface of airway epithelial cells is indispensable to initiating MP colonization and infection [45,46,47]. These results suggest that GOS could be used in clinics to prevent (MR)MP carriage in vulnerable patients—for example, those with hypogammaglobulinemia or sickle cell anemia, who are particularly susceptible to MP pulmonary disease and/or extrapulmonary manifestations [48,49,50,51,52,53]. 

Contrary to our findings, FOS structures have been shown to prevent the adhesion of *E. coli, Clostridium difficile*, Bacteroides and *Listeria monocytogenes* to intestinal epithelial cells in vitro [54,55,56,57]. Since these studies show that the adhesion of both Gram-positive and Gram-negative bacteria can be inhibited, the lack of anti-adhesive effects of FOS in our MP cultures does not seem to be due to restriction to a certain bacteria class. However, it may be speculated that FOS specifically target structures expressed on intestinal, but not respiratory, epithelial cells or target bacterial adhesins specific to intestinal epithelial cells.

By preventing the adhesion of MP and thus decreasing the number of MP/MRMP on the epithelial cell surface, GOS also significantly prevent inflammation. We and others showed that, following the engagement of TLRs on A549 cells, MP induce the production of the pro-inflammatory cytokines IL-6 and IL-8 [58,59]. However, the amounts of IL-6 and IL-8 were also significantly decreased when 4% GOS was added to A549 cultures with adherent MP/MRMP, which points to a different mechanism of anti-inflammatory action of GOS. While these cytokines are important to recruiting immune cells to control infection, overzealous amounts of these cytokines are also linked to the (histo)pathology of the lungs [1,60]. Curbing inflammation via GOS treatment will protect from damage to the lung structure and thereby protect lung function. Interestingly, no such strong dampening of inflammatory IL-6 and IL-8 production was observed in ERY-treated cultures. This finding contrasts with several clinical and experimental observations that point towards the anti-inflammatory effects of macrolides [61,62,63]. However, differences may be related to the doses, pathogens and timing of ERY application. For example, some in vitro studies with bronchial epithelial cells used a high dose of ERY (0.1–10 μg/mL), which can significantly block the *Haemophilus influenzae* endotoxin-induced release of IL-6 and IL-8, while our experiments with A549 cells using a low concentration of ERY (0.004 μg/mL) did not inhibit the MP/MRMP-induced secretion of IL-6 and IL-8 [63]. Additionally, the timing of treatment may be an important factor, as Jang et al. pretreated the A549 epithelial cells for 72 h with ERY before adding the pathogenic stimulus and found reduced levels of pro-inflammatory cytokines [61]. 

Overall, GOS have anti-adhesive and bactericidal effects on MP and MRMP, resulting in the reduced infection of airway epithelial cells and reduced cytokine secretion, which indicate the potential of these compounds for use as anti-infective and anti-microbial agents for the treatment of MP and MRMP infections and for reducing the antibiotic overuse in clinical practice. However, studies in mice are needed to test the efficacy of GOS in resolving MRMP respiratory infections in an in vivo setting. While the oral administration of GOS could be effective for the treatment or prevention of respiratory infections [64], administration via the airways may be a more effective and direct strategy for targeting respiratory pathogens. Data from a recent study in calves demonstrated that the intranasal administration of GOS resulted in a reduction in respiratory infections with *M. haemolytica* as well as alleviated pulmonary inflammation [27], which implies that GOS as an inhalation therapy for the treatment of respiratory bacterial infections can be feasible. 

## Figures and Tables

**Table 1:** The MIC values of GOS and FOS for MP and MRMP.

	MIC Values							
Strains	MP				MRMP			
	M129	FH	A103	H010	A58	M688/98	T79	P05/132
**GOS (%)**	4	4	4	4	4	4	4	4
**FOS (%)**	16	16	8	16	16	16	16	16
**ERY (µg/mL)**	0.032	0.032	0.032	0.032	256	256	256	256

MIC, minimum inhibitory concentration; MP, *Mycoplasma pneumoniae*; MRMP, macrolide-resistant *Mycoplasma pneumoniae*; GOS, galacto-oligosaccharides; FOS, fructo-oligosaccharides; ERY, erythromycin.

## Data Availability

The data presented in this study are available upon reasonable request from the corresponding author.

## Supplementary Materials

The following supporting information can be downloaded at: www.mdpi.com/article/10.3390/pathogens12050659/s1, Figure S1: GOS and FOS are not cytotoxic to epithelial cells; Figure S2: ERY does not possess anti-adhesive or anti-inflammatory capacity nor affects MP viability; Figure S3: FOS do not inhibit MP adhesion to epithelial cells.
