# Supplementary material for: Galacto-Oligosaccharides as an Anti-Infective and Anti-Microbial Agent for Macrolide-Resistant and -Sensitive Mycoplasma pneumoniae
Source: Pathogens. 2023 Apr 28;12(5):659. doi: 10.3390/pathogens12050659 (PMC10224470; doi:10.3390/pathogens12050659)
Supplement: Supplementary file 1 [file pathogens-12-00659-s001.zip › pathogens-2349639-supplementary.pdf]

Supplement materials

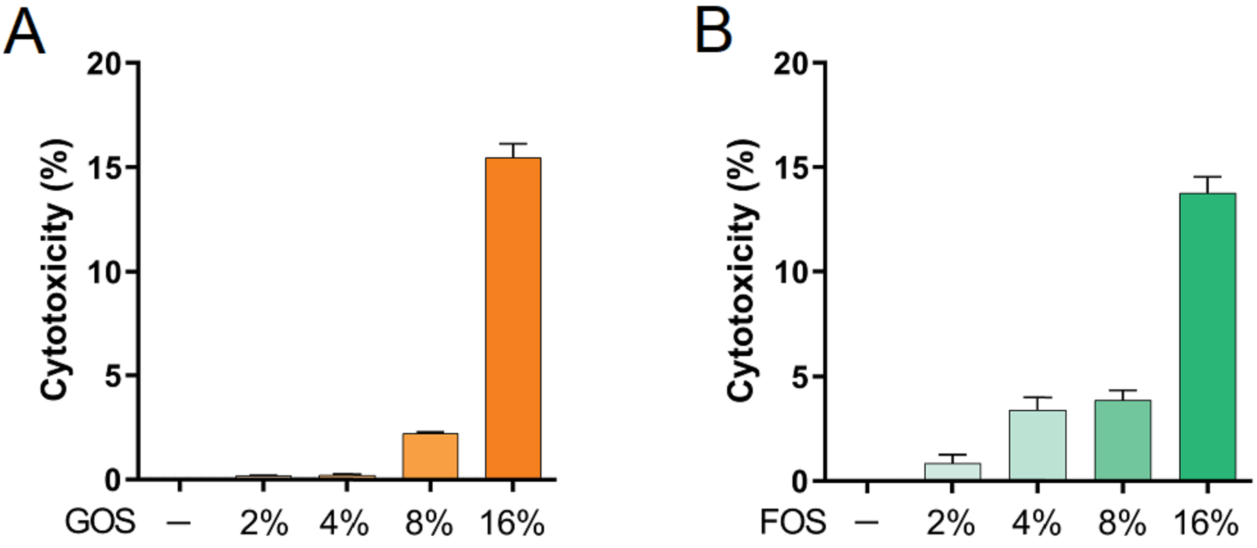

Figure S1. GOS and FOS are not cytotoxic to epithelial cells.
Respiratory epithelial cells were cultured with indicated concentrations of GOS for 24 h, thereafter the potential toxicity of GOS (**A**) or FOS (**B**) to A549 cells was measured by assessing the cell viability using the lactate dehydrogenase (LDH) assay. Bars represent cytotoxicity.

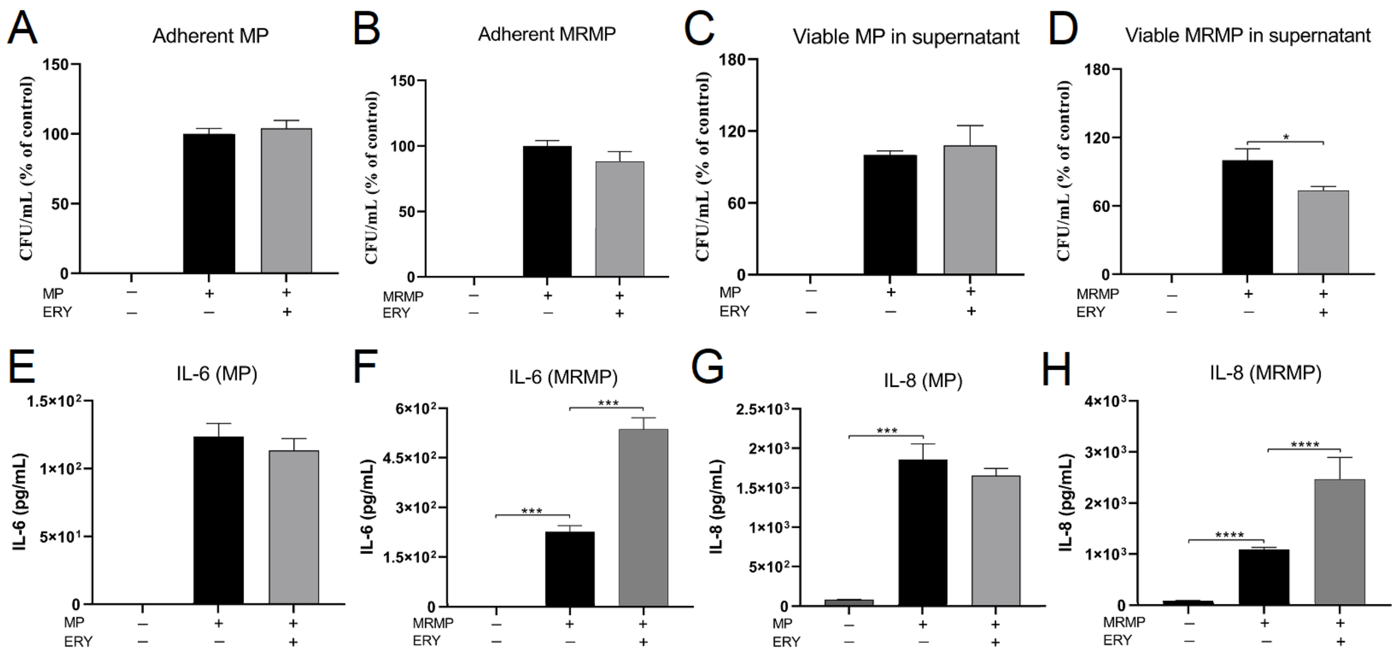

Figure S2. ERY does not possess anti-adhesive or anti-inflammatory capacity nor affect MP viability.
MP or MRMR were first adhered to respiratory epithelial cells by co-culturing for 4 h. Thereafter, co-cultures were treated with ERY, and to control cultures medium was added. After overnight culture, the supernatants were collected and epithelial cells were lysed. Both, cells (**A**,**B**) and culture supernatants (**C**,**D**) were inoculated onto PPLO agar plates and CFUs were determined. The mean CFU/mL ± SEM are shown as % of control. IL-6 (**E**,**F**) and IL-8 (**G**,**H**) levels in culture supernatants were measured by ELISA. Bars represent the mean cytokine concentration ± SEM. * $p < 0.05$, *** $p < 0.001$ and **** $p < 0.0001$ (One-way ANOVA followed by Dunnett's multiple comparison test was used for statistical analysis).

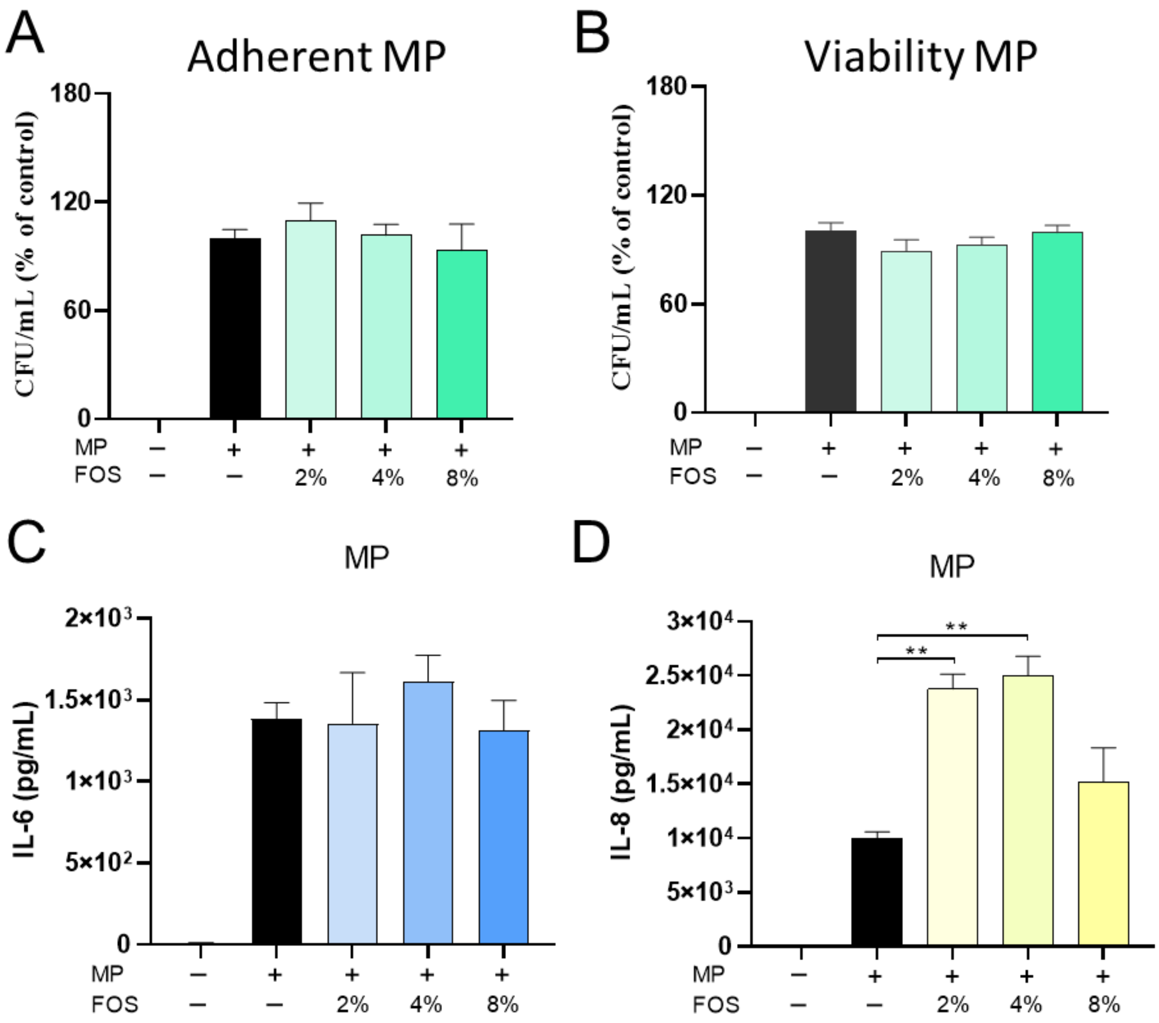

Figure S3. FOS does not inhibit MP adhesion to epithelial cells.
MP was cultured on epithelial cells in the presence of indicated concentrations of FOS. Supernatants were collected 24h later and epithelial cells were lysed. Both, cells (**A**) and culture supernatant (**B**) were inoculated onto PPLO agar plates and CFUs were determined. The mean CFU/mL ± SEM are shown as % of control. Additionally, IL-6 (**C**) and IL-8 (**D**) levels in supernatants were measured by ELISA. ** $p < 0.01$ (One-way ANOVA followed by Dunnett's multiple comparison test was used for statistical analysis).
